# Discovering therapeutic possibilities for polycystic ovary syndrome by targeting *XIST* and its associated ceRNA network through the analysis of transcriptome data

**DOI:** 10.1038/s41598-024-56524-1

**Published:** 2024-03-14

**Authors:** Elahe Berenji, Ali Valipour Motlagh, Marziyeh Fathi, Maryam Esmaeili, Tayebeh Izadi, Parsa Rezvanian, Maryam Zanjirband, Zahra Safaeinejad, Mohammad Hossein Nasr-Esfahani

**Affiliations:** 1 ACECR Institute of Higher Education (Isfahan Branch), Isfahan, Iran; 2 Department of Cellular Biotechnology, Cell Science Research Center, Royan Institute for Biotechnology, ACECR, P.O. Box 816513-1378, Isfahan, Iran

**Keywords:** Polycystic ovary syndrome, *XIST*, ceRNA network, Biomarker, Drug repositioning, Cell biology, Computational biology and bioinformatics, Metabolic disorders, Reproductive disorders

## Abstract

Long non-coding RNA (lncRNA) regulates many physiological processes by acting as competitive endogenous RNA (ceRNA). The dysregulation of lncRNA X-inactive specific transcript (*XIST*) has been shown in various human disorders. However, its role in the pathogenesis of polycystic ovary syndrome (PCOS) is yet to be explored. This study aimed to explore the underlying mechanism of *XIST* in the pathogenesis of PCOS, specifically through dataset functional analysis. GEO PCOS datasets including RNA-seq, microarray, and miRNA-seq in granulosa cells (GCs) and blood, were examined and comprehensively analyzed. Enrichment analysis, ROC curve constructions, lncRNA-miRNA-mRNA interaction network analyses, and qRT-PCR validation were performed followed by a series of drug signature screenings. Our results revealed significant dysregulation in the expression of 1131 mRNAs, 30 miRNAs, and *XIST* in GCs of PCOS patients compared to healthy individuals. Of the120 *XIST*-correlated upregulated genes, 25 were enriched in inflammation-related pathways. Additionally, 5 miRNAs were identified as negative regulators of *XIST*-correlated genes. Accordingly, a ceRNA network containing *XIST*-miRNAs-mRNAs interactions was constructed. Furthermore, 6 genes, including *AQP9, ETS2, PLAU, PLEK, SOCS3,* and *TNFRSF1B* served as both GCs and blood-based biomarkers. By analyzing the number of interactions among *XIST*, miRNAs, and mRNAs, we pinpointed *ETS2* as the pivotal gene within the ceRNA network. Our findings reveal a novel *XIST*- hsa-miR-146a-5p, hsa-miR-144-3p, and hsa-miR-1271-5p-*ETS2* axis that comprehensively elucidates the *XIST*-associated mechanism underlying PCOS onset. qRT-PCR analysis further confirmed the, overexpression of both *XIST* and *ETS2* . Furthermore, our results demonstrated that *XIST* and *ETS2* were correlated with some assisted reproductive technologies outcomes. Finally, we identified two novel compounds including, methotrexate/folate and threonine using drug–gene interaction databases for PCOS management. These findings provide novel insights into the molecular etiology, diagnosis, and potential therapeutic interventions for PCOS.

## Introduction

Polycystic ovary syndrome (PCOS) constitutes a chronic reproductive and metabolic disorder, affecting up to 20% of women within their reproductive age^1,2^. Originally described by Stein and Leventhal in 1935, PCOS is diagnosed based on the manifestation of at least two out of three Rotterdam criteria including hyperandrogenism, oligo-or anovulation, and ovaries with polycystic morphology^3^. Moreover, a considerable percentage of women suffering from PCOS, also endure other complications such as obesity, hypertension, type 2 diabetes, cardiovascular disease, and mental health issues such as anxiety and depression^4^. Despite this wide range of complications associated with PCOS, its genetic, epigenetic, and molecular etiology remain unclear. In light of previous researches, it is suggested that the epigenetic pattern of individuals with PCOS may have been altered by intrauterine and infancy history as well as their lifestyle^5^.

Dysregulated expression of different non-coding RNAs and their corresponding target genes have been reported in serum, follicular fluid (FF), and granulosa cells (GCs) of women suffering from PCOS^5^. Long non-coding RNAs (lncRNAs) and microRNAs (miRNAs), which constitute the most extensively studied classes of non-coding RNAs, exhibit mutual fundamental interaction to regulatethe transcriptional levels of numerous genes at the post-transcription level^6^. Since long non-coding RNAs contain microRNA Response Elements (MREs), they can act as sponge/decoy factors to inhibit the repression of miRNA-target mRNAs^7^. Accordingly, through the intricate lncRNA-miRNA-mRNA regulatory network, LncRNAs can play a significant role in the progression and pathogenesis of a wide range of diseases^8^.

LncRNA X-inactive specific transcript *(XIST*), located on chromosome Xq13.2, is involved in the random X chromosome inactivation observed in female cells of placental mammals^9^. Based on clear and convincing evidence, *XIST* functions as a principal regulator of cell growth, apoptosis, and development^10^. More recently, abnormal expression of *XIST* has been observed in a variety of human disorders, including different types of tumors^11^, gestational diabetes mellitus^12^, diabetic neuropathy, autoimmune disorders, and neurological diseases^13^. Although previous findings have unveiled the potential role of *XIST* in various human diseases, its specific role within the context of PCOS remains inadequately investigated. Therefore, in the current study, we aimed to explore the underlying mechanism of *XIST* contribution in the pathogenesis of PCOS using analysis of high-throughput available data and assessing both *XIST* and its key target gene, *ETS2,* in granulosa cells (GCs) of individuals afflicted with PCOS by qRT-PCR. Finally, novel therapeutic compounds were identified with a drug repositioning approach.

## Results

### Identification of differentially expressed genes (DEGs) in granulosa cells (GCs) between individuals with PCOS and controls

**Figure 1:**
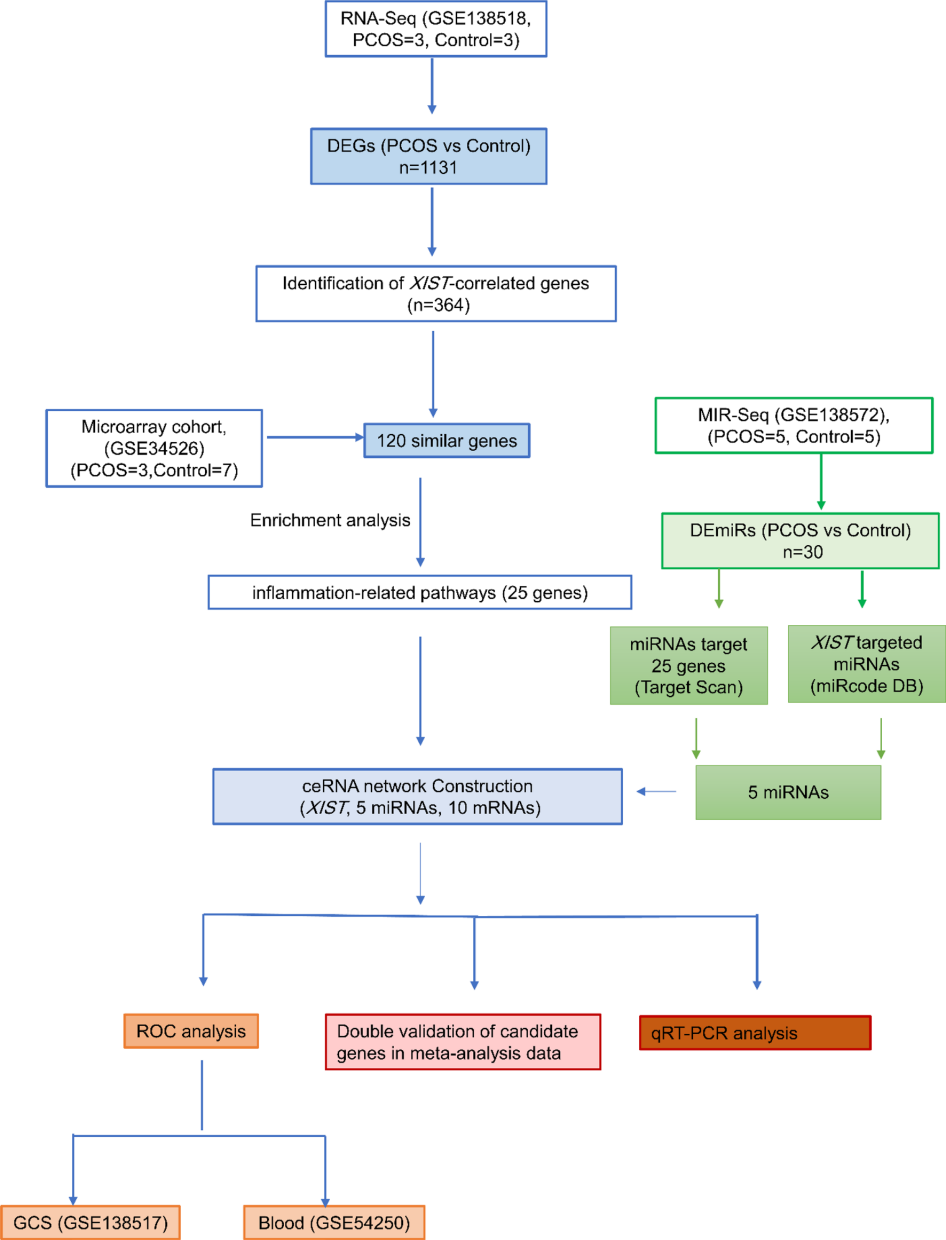
Flowchart for bioinformatics analysis of publicly available RNA sequencing datasets.ceRNA, Competitive endogenous RNA; DEG, Differential Expression Gene; GCs, Granulosa cells; GEO, Gene Expression Omnibus; *XIST*, lncRNA X-inactive specific transcript; PCOS, Polycystic ovary syndrome; ROC, Receiver Operating Characteristic; miRNAs, microRNAs; lncRNAs, long non-coding RNAs; seq, sequencing.

**Figure 2:**
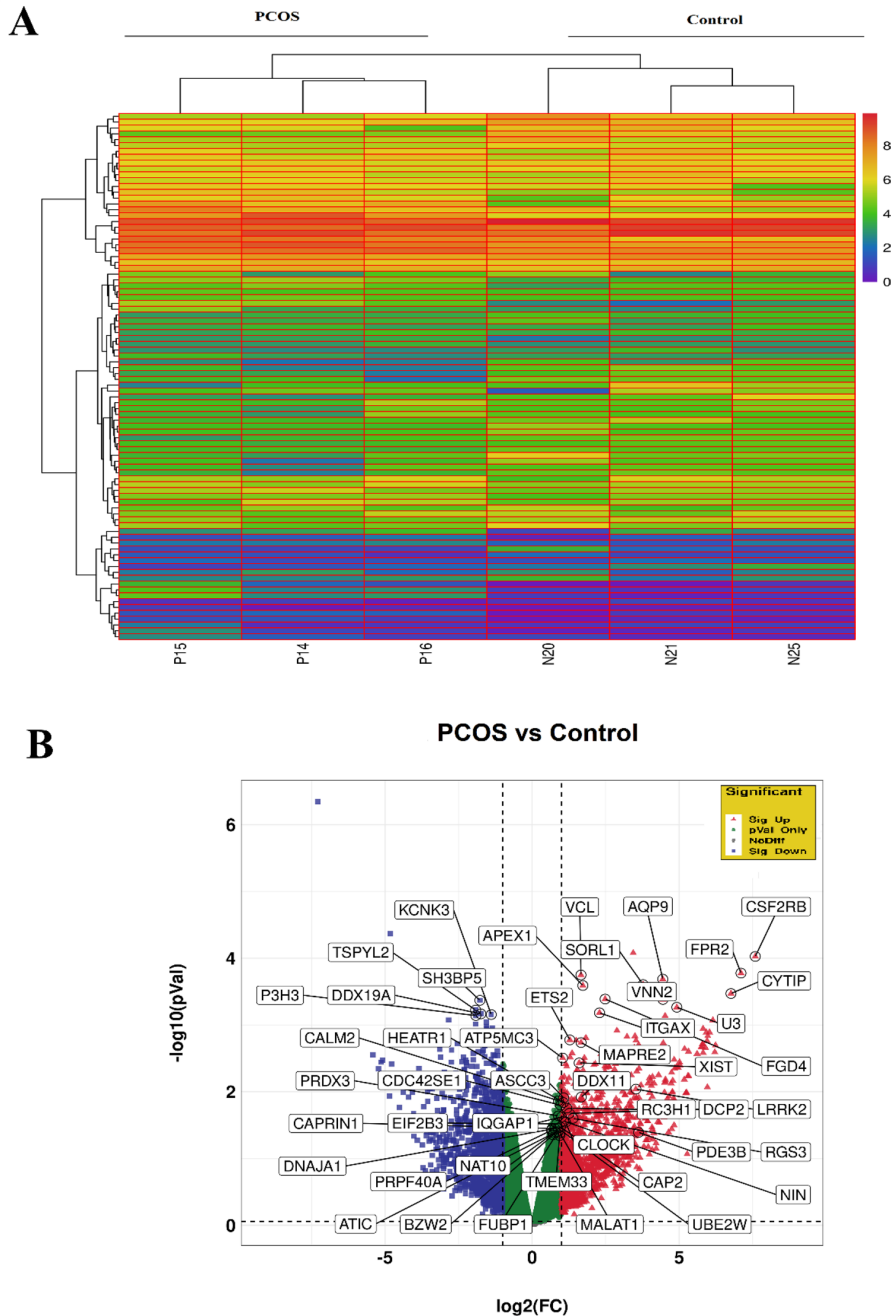
Differential gene expression profile of individuals with PCOS vs. controls. (**A**) Heatmap plot depicting differentially expressed genes based on the GSE138518 dataset. (**B**) Volcano plot displaying significantly up-regulated genes with red dots and significantly down-regulated genes with blue dots. PCOS, Polycystic ovary syndrome.

The flowchart outlining the methodology of this study is presented in Fig. 1. To detect significant alterations in lncRNAs and mRNAs among PCOS women, differential expression analyses were performed on 3 PCOS and 3 control samples based on GSE138518, using the edgeR package, with thresholds set at *FDR* < 0.05 and − 1 <|log2FC|≥ 1. The hierarchical clustering indicated apparent differences in expression patterns between PCOS and control samples (Fig. 2A). Furthermore, a volcano plot was generated using ggplot2 in the R platform to visually depict differential gene expression between PCOS and control GCs (Fig. 2B). Our findings revealed that a total of 1131 genes were identified as DEGs, consisting of 571 up-regulated and 560 down-regulated genes in PCOS compared to control GCs. As shown in Fig. 2B, *XIST* as the conserved lncRNA, was detected among up-regulated genes in women suffering from PCOS..

### Identification of *XIST*-correlated genes in PCOS

**Figure 3:**
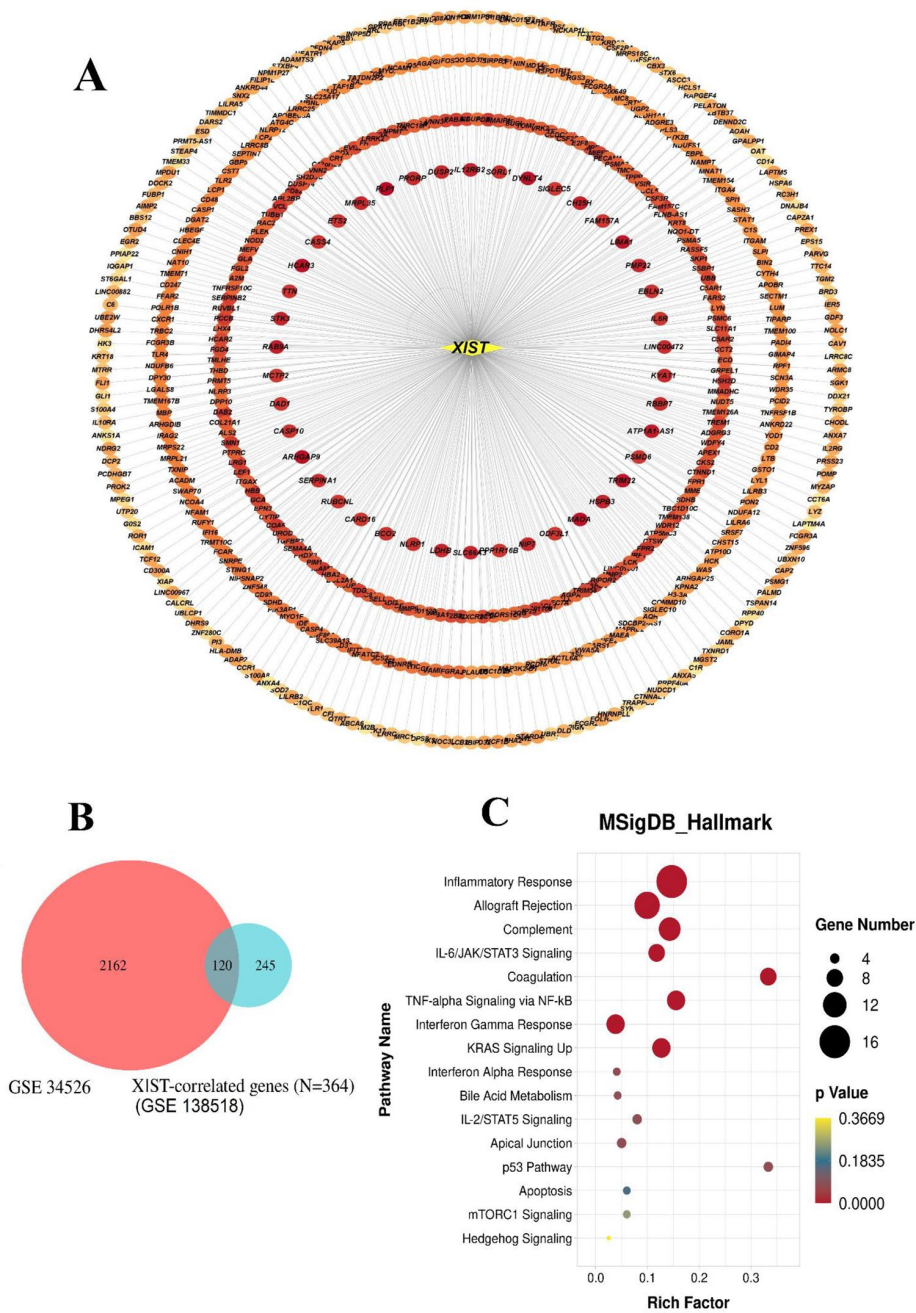
The co-expression network map between *XIST* and differentially expressed mRNA, validation of the expression levels of genes, and enrichment analysis. (**A**) *XIST*-correlated genes were presented by Cytoscape. Dark red indicates a stronger correlation between *XIST* and DEMs. (**B**) The Venn diagram of differentially expressed genes based on GSE34526 and *XIST*-correlated genes with Pearson’s r > 0.7. (**C**) Bubble chart of the Molecular Signatures Database enrichment analysis of 120 *XIST*-correlated genes. *XIST*, lncRNA X-inactive specific transcript; DEMs, differentially expressed miRNAs; lncRNAs, long non-coding RNAs.

To identify genes more likely to be regulated by *XIST* in GCs, a Pearson correlation test was applied, examining the correlation between the expression of *XIST* and all significant DEGs. The results indicated that 856 genes exhibited correlations with *XIST*, with 538 genes showing a positive correlation coefficient > 0.5 with a *p* *value* < 0.05 and 318 genes exhibiting a negative correlation coefficient < − 0.5 with a *p* *value* < 0.05. Specifically, genes with a correlation coefficient > 0.5 and *p* *value* < 0.05 were designated as candidate genes. To visualize these correlations, a circular diagram was created using Cystoscope software (Fig. 3A). The correlation values for each gene can be found in Supplementary Data 1. Out of the initial 538 genes, 364 genes with Pearson’s r > 0.7 were selected for further analysis. For additional verification, the expression levels of these 364 candidate genes were evaluated in the microarray GSE34526. The results illustrated as a Venn diagram (Fig. 3B and Supplementary Fig. S1) showed that 120 genes, including *XIST* itself*,* were significantly up-regulated in PCOS samples of validation cohorts compared to control GCs. Consequently, 120 *XIST*-correlated genes were selected for the subsequent phase of this study.

### *XIST*-correlated genes can play a vital role in the development of PCOS via inflammation-related pathways

To identify *XIST*-related pathways, an enrichment analysis of 120 *XIST*-correlated genes was performed utilizing the Molecular Signatures Database (MSigDB) and Enrichr software. The results of this analysis revealed that the 120 putative target genes of *XIST* could be enriched in the Inflammatory Response (Adjusted *p* value = 2.18E−12), Allograft Rejection (Adjusted *p* value = 3.71E-09), Complement (Adjusted *p* value = 3.64E−07), IL-6/JAK/STAT3 Signaling (Adjusted *p* value = 4.18E−07), Coagulation (Adjusted *p* value = 1.18E−05), TNF-alpha Signaling via NF-kB (Adjusted *p* value = 1.34E−05), Interferon Gamma Response (Adjusted *p* value = 1.34E−05), and KRAS Signaling Up (Adjusted *p* value = 1.34E−05) (Fig. 3C and Supplementary Table S2). Considering the well-established role of Inflammatory Response, IL-6/JAK/STAT3, and TNF-alpha Signaling via the NF-kB signaling pathways in the progression of PCOS^14,15^, all three pathways and their 25 associated genes including *LYN; CSF3R; CD82; AQP9; C5AR1; FPR1; NOD2; TNFRSF1B; LCK; IRF1; NAMPT; NLRP3; FFAR2; LCP2; TLR2; HBEGF; SOCS3; CSF3R; STAT1; LTB; A2M; SERPINB2; PLAU; PLEK; ETS2* were selected for further investigation. Importantly, these pathways all had an FDR < 0.01 and duplicate genes within these three pathways, were counted only once.

### Distinguishing DEmiRs between individuals with PCOS and controls and, ceRNA network construction

**Figure 4:**
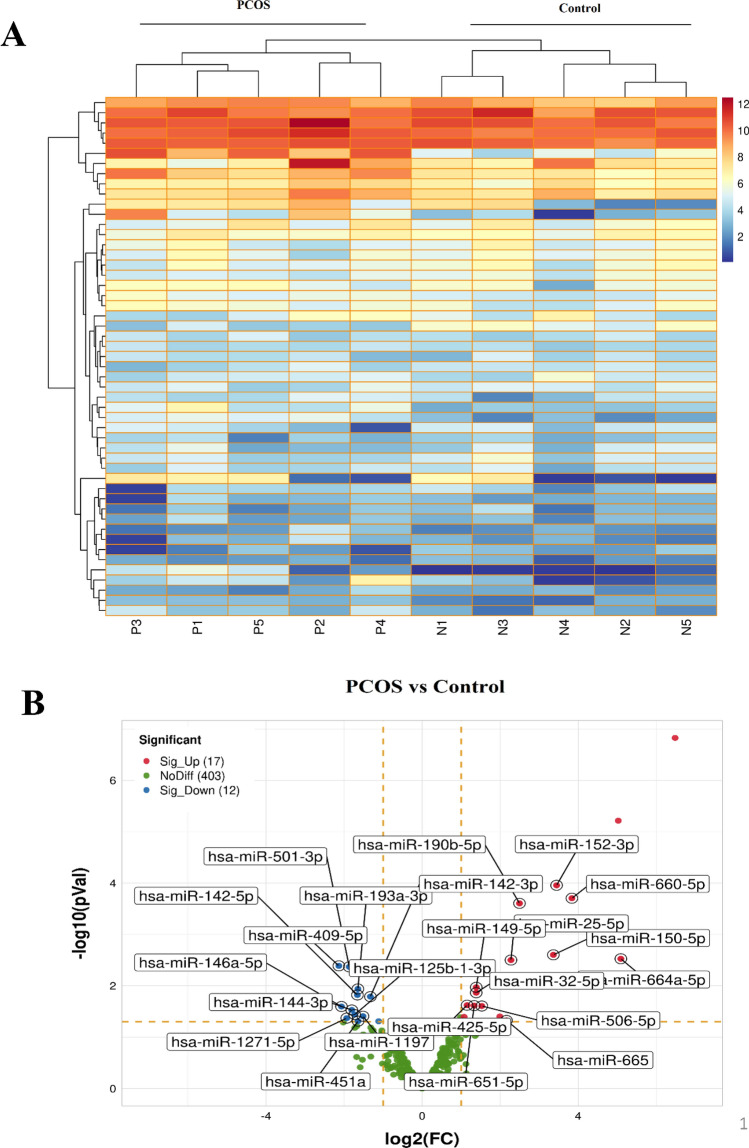
Identification of DEmiRs between individuals with PCOS and controls. (**A**) Heatmap plot depicting the miRNAs expression profile of GSE138572 in individuals with PCOS vs. controls. (**B**) Volcano plot indicating 17 up-regulated and 13 down-regulated miRNAs in individuals with PCOS vs. controls. DEmiRs, differential expression of microRNAs; PCOS, Polycystic ovary syndrome.

**Figure 5:**
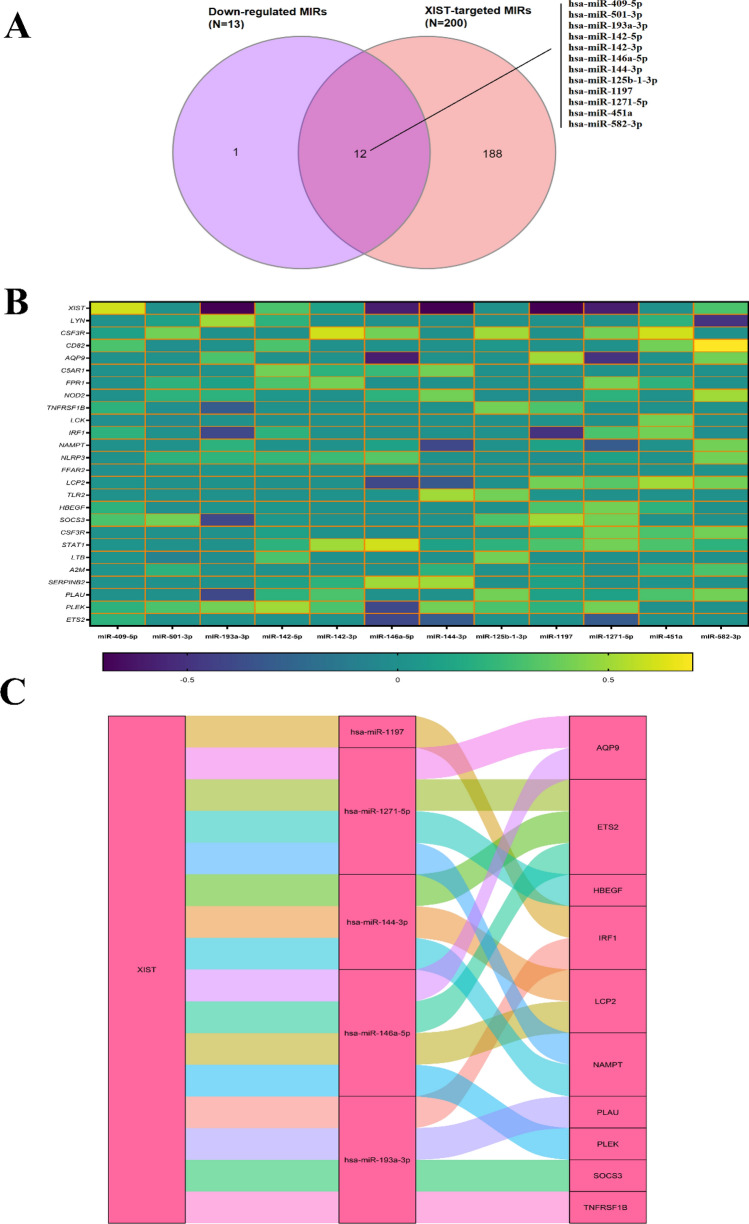
Construction of the ceRNA network for *XIST*-miRNAs-mRNAs. (**A**) The overlapping DE miRs and *XIST*-targeted miRNAs. (**B**) Pearson correlation analysis of the 12 miRNAs with *XIST* and 25 *XIST*-related genes. Light colors represent a positive correlation, while dark colors represent a negative correlation. The genes with no significant correlation were shown with zero correlation. (**C**) Sankey diagram indicating interactions between *XIST* and their matched miRNAs and mRNAs. Each rectangle represents a gene, and the connection degree of each gene is visualized based on the size of the rectangle. ceRNA, Competitive endogenous RNA; DEmiRs, differential expression of microRNAs; miRNAs, microRNAs.

Differential miRNA expression analysis (DemiRs) on 5 PCOS samples and 5 control samples related to GSE138572, showed significant differential expression in 30 miRNAs (Fig. 4A). Out of these 30 miRNAs, 17 miRNAs were up-regulated and 13 miRNAs were down-regulated according to the criteria established in this study (Fig. 4B). To gain insight into the interaction between *XIST*, DemiRs, and *XIST*-correlated genes, the *XIST*-miRNA-mRNA ceRNA network was constructed. To this aim, *XIST-*targeted miRNAs were obtained from the miRcode database. According to this database, *XIST* could be potentially targeted by 200 miRNAs. However, only 12 miRNAs overlapped with the down-regulated DemiRs, qualifying them as candidates for our network (Fig. 5A). Furthermore, the interaction between the 12 miRNAs and the 25 *XIST*-correlated genes was corroborated using the TargetScan database. Additionally, the negative correlation pattern between the expression levels of *XIST* and *XIST*-correlated genes with candidate miRNAs was confirmed (Fig. 5B). Finally, the ceRNA network was constructed by *XIST*, 5 miRNAs including hsa-miR-1197, hsa-miR-193a-3p, hsa-miR-144-3p, hsa-miR-1271-5p and hsa-miR-146a-5p and 10 mRNAs including *AQP9, ETS2, HBEGF, IRF1, LCP2, NAMPT, PLAU, PLEK, SOCS3,* and *TNFRSF1B* (Fig. 5C). Intriguingly, among the 10 evaluated mRNAs, only *ETS2* was found to be targeted by 3 candidate miRNAs namely, hsa-miR-146a-5p, hsa-miR-144-3p, and hsa-miR-1271-5p.

### Meta-analysis of gene expression profiles of PCOS and validation of *XIST* and *ETS2* upregulation through qRT-PCR

**Figure 6:**
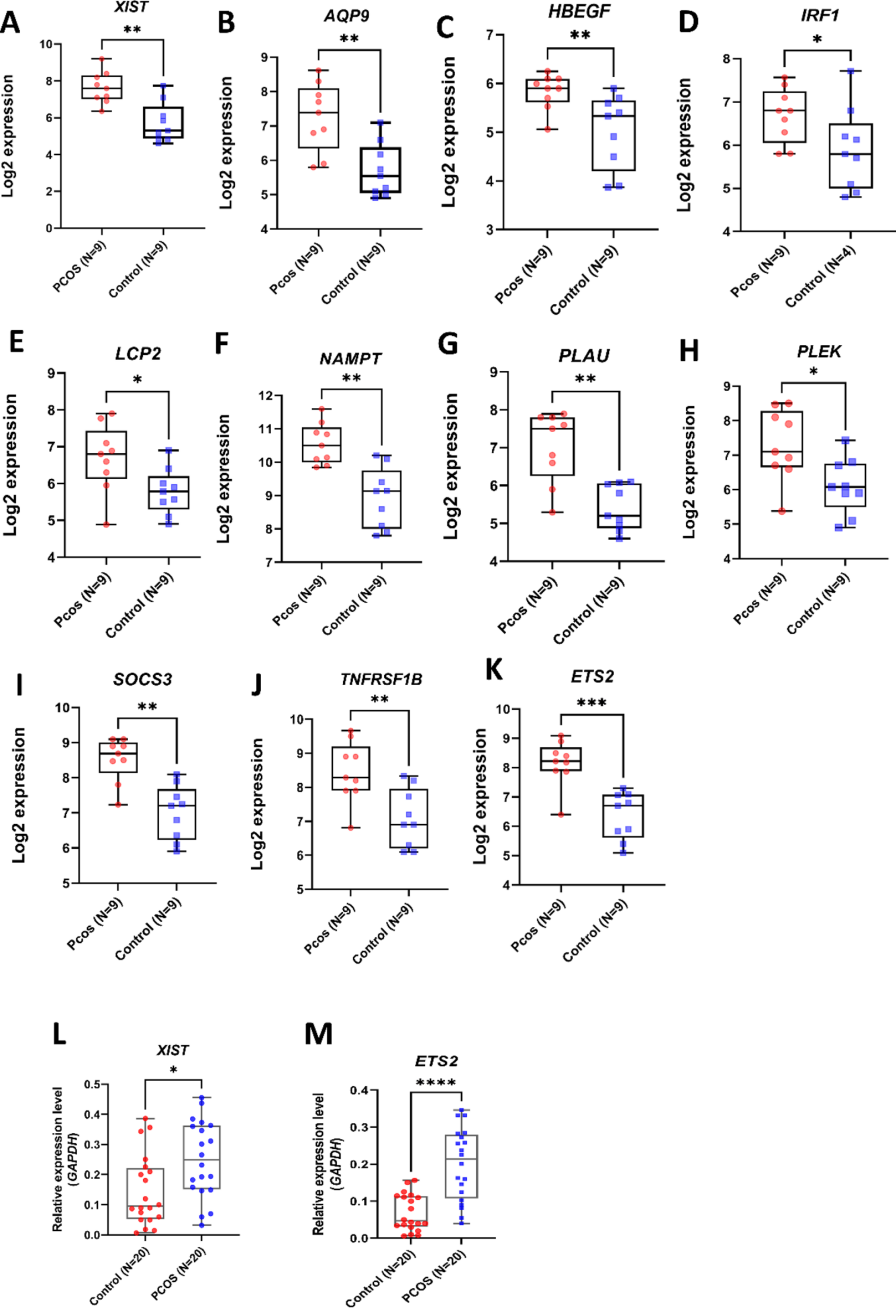
Meta-Analysis and qRT-PCR Validation. (**A**–**K**) Elevated levels of *XIST* and ten associated genes evaluation based on meta-analysis data. (**L**–**M**) Boxplots representing qRT-PCR results for *XIST* and *ETS2* expression in PCOS (n = 20) vs control (n = 20) GCs.

To assess the expression levels of our candidate genes across various studies, we aggregated samples from two independent public studies and constructed a meta-analysis dataset (see Materials and Methods). Our results revealed significant changes in 2172 genes in PCOS granulosa cells, with 1189 genes upregulated and 983 genes downregulated, all meeting the predefined criteria (Supplementary Fig. S1). In the subsequent analysis, we evaluated the expression levels of our ceRNA axis. Our findings confirmed elevated levels of both *XIST* and ten *XIST*-related genes in PCOS granulosa cells (Fig. 6A-K). To validate the accuracy of these cohort results and enhance the reliability of our findings, *XIST* expression and *ETS2* expression in the GCs of 20 PCOS and 20 control women were measured using qRT-PCR. The results revealed that both *XIST* (Fig. 6L) and *ETS2* (Fig. 6M) were significantly up-regulated in the PCOS group compared the control group.

### Correlation between baseline characteristics or assisted reproductive technologies (ARTs) outcomes of PCOS individuals with *XIST* and its correlated gene *ETS2*

A Pearson correlation test was performed to evaluate the correlation between *XIST* and *ETS2* with baseline parameters or ART outcomes of PCOS women (Table 1). We found that there is a significant positive correlation between the expression levels of *XIST* and the number of germinal vesicle-stage oocytes (GV oocytes) (r = 0.5113, *p* = 0.0178). Meanwhile, over-expressed *XIST* showed a significant negative correlation with the number of MII oocytes (*p* = 0.0446, r = − 0.432). In line with *XIST*, up-regulated *ETS2* in PCOS women indicated a significant direct correlation with the percentage of GV (r = 0.654, *p* = 0.0389) and a significant reverse correlation with the percentage of MII oocytes (r = − 0.456, *p* = 0.0214).

**Table 1:** Clinical characteristics of polycystic ovarian syndrome (PCOS) and control individual.

Data	Control (n = 20)	PCOS (n = 20)	*P* value
Age (years)	32.09 ± 4.2	31.82 ± 4.3	0.9123
BMI (kg/m^2^)	21.88 ± 1.28	21.92 ± 1.37	0.9812
LH (mUI/mL)	4.44 ± 1.3	7.18 ± 2.7	0.0031*
FSH (mUI/mL)	5.37 ± 1.7	5.04 ± 2.5	0.5123
LH/FSH ratio	1.2 ± 0.6	1.9 ± 2.2	0.5456
AMH (ng/mL)	1.88 ± 1.15	7.22 ± 4.11	0.0158*
T(nmol/L)	19.5773 ± 2.36883	26.3125 ± 5.35280	0.00136
COC	16.79 ± 10.4	17 ± 6.7	0.5753
%GV	17.2 ± 26.2	11.79 ± 15.23	0.5423
%MII	82.8 ± 26.2	88.2 ± 15.2	0.5156
%Degenerated Oocyte	12.38 ± 20.5	11.65 ± 13.71	0.9623
%2PN	68.39 ± 22.6	66.39 ± 19.5	0.5423
%Blastocyst	31.49 ± 34.31	39.14 ± 30.57	0.4123
%Cleavage	97.92 ± 7.2	100	0.3124
Number of Useable Embryo	5.0 ± 4.2	5.57 ± 4.65	0.7233

*BMI* body mass index, *LH* luteinizing hormone, *FSH* follicle-stimulating hormone, *T* total testosterone, *AMH* anti-mullerian hormone, *COC* cumulus oocyte complex, *GV* germinal vesicle, *MII* Mature oocytes, 2PN fertilized oocytes with two primary pronucleus.

*p < 0.05.

### XIST and XIST- correlated genes could be considered diagnostic markers

**Figure 7:**
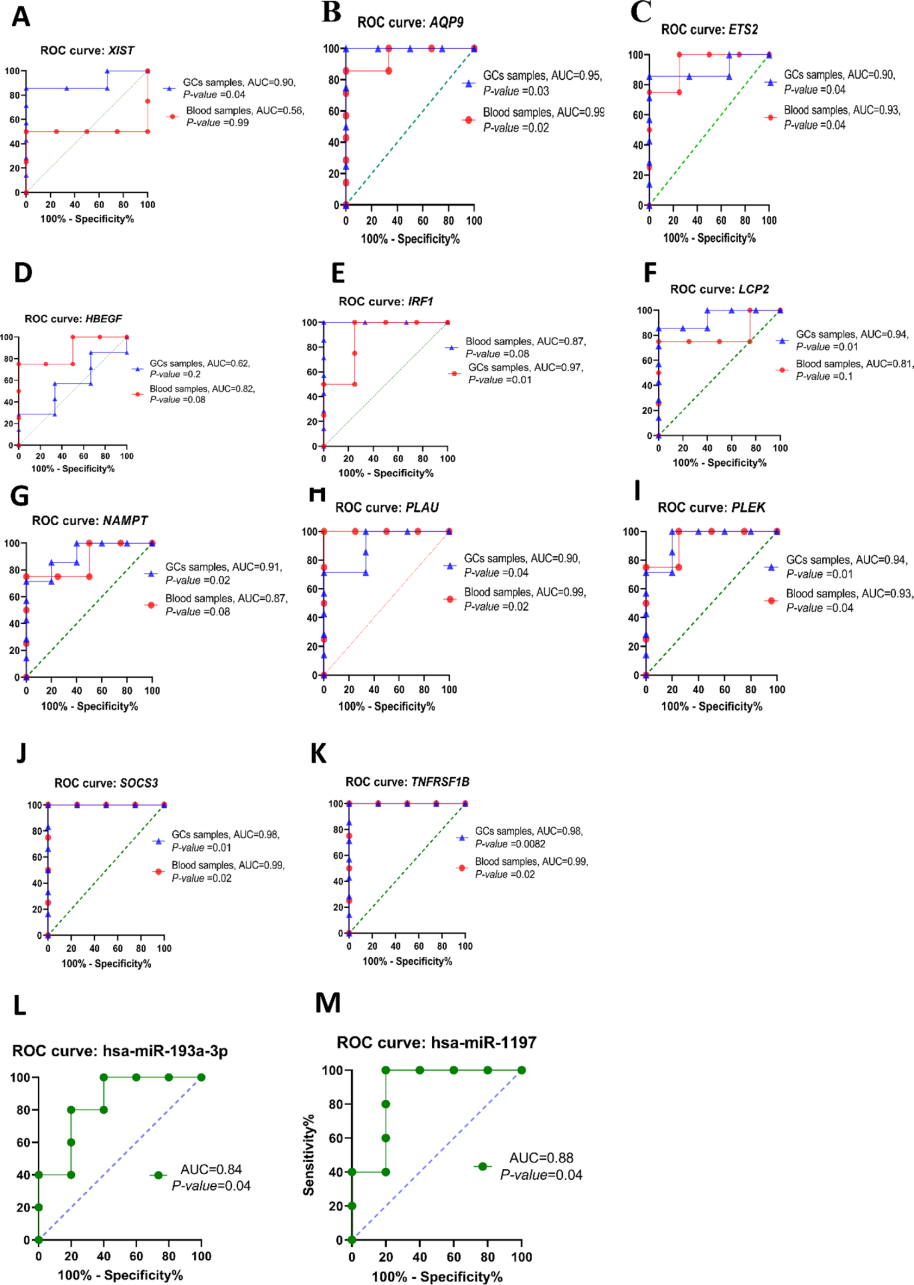
ROC curve analyses of *XIST*, *XIST*-related genes, and *XIST*-related miRNAs. (**A**–**K**) ROC curves for *XIST* and 10 *XIST*-correlated genes based on the normalized expression data extracted from GCs samples (GSE34562) and blood (GSE54248) samples. The genes with AUC > 0.9 have better diagnostic efficacy for the identification of PCOS individuals. (**L**–**M**) ROC plots of selected miRNAs based on GSE138572. *XIST*, lncRNA X-inactive specific transcript; miRNAs, microRNAs; ROC, Receiver Operating Characteristic; GCs, Granulosa cells; AUC, Area under the ROC Curve.

The expression levels of *XIST*, 10 mRNAs, and 5 miRNAs obtained from the ceRNA network were subjected to ROC analysis to assess their ability to distinguish PCOS from control individuals using the GSE34562 study. Our results exhibited that the AUC of *XIST* (0.90, *p* value = 0.04) could effectively distinguish PCOS from the control population, signifying its potential as a diagnostic biomarker in GCs (Fig. 7A). The AUC for the candidate genes was as follows: (*AQP9* = 0.95, *p* *value* = 0.03), (*ETS2* = 0.90, *p value* = 0.04), (*HBEGF* = 0.68, *p value* = 0.2), (*IRF1* = 0.99, *p* *value* = 0.02), (*LCP2* = 0.94, *p value* = 0.01), (*NAMPT* = 0.91, *p value* = 0.02), (*PLAU* = 0.90, *p* *value* = 0.04), (*PLEK* = 0.94, *p value* = 0.01), (*SOCS3* = 0.99, *p value* = 0.01) and (*TNFRSF1B* = 0.99, *p* *value* = 0.001). The results indicated that the average of AUC, without considering *HBEGF,* was greater than 0.9, signifying the potential of these genes to be employed as biomarkers in GCs (Fig. 7B–K). The diagnostic value of five miRNAs was obtained through ROC analysis based on GSE138572. The outcome showed that only hsa-miR-1197 with AUC = 0.88, *p *value = 0.04 and hsa-miR-193a-3p with AUC = 0.84, *p* value = 0.04 could be suggested as potential biomarkers in GCs (Fig. 7 L-M). To further validate the diagnostic potential of the selected genes as biomarkers, the blood microarray GSE54248 study, which includes 4 PCOS and 4 control samples was utilized. The expression levels of candidate genes were evaluated, and subsequently, ROC analysis was performed. The findings indicated that *AQP9, ETS2, LCP2, NAMPT, PLAU, PLEK, SOCS3*, and *TNFRSF1B* were significantly overexpressed in blood samples from individuals with PCOS when compared to control samples. However, the expression levels of *XIST*, *HBEGF*, and *IRF1* were not significantly altered ((Supplementary Fig. S3). The ROC analysis results showed that *AQP9* (Fig. 7B)*, ETS2* (Fig. 7C)*, PLAU* (Fig. 7H)*, PLEK* (Fig. 7I)*, SOCS3* (Fig. 7J)*,* and *TNFRSF1B* (Fig. 7K) genes can distinguish between PCOS and control samples with AUC values greater than 0.9. Therefore, we propose that these 6 candidate genes may be used as potential diagnostic biomarkers for PCOS in both tissues and blood samples.

### Exploring drug candidates through predictive datasets

The identified genes in individuals with PCOS were uploaded to multiple datasets, includingGeneCards , DRUGBANK, TTC (Therapeutic Target Database), DGidb (Drug Gene Interaction Database), and GEO , to identify potential drugs that target these genes (Table 2).

**Table 2:** Pearson correlation analysis of *XIST* and *ETS2* with baseline characteristics and ARTs outcomes of subjects with PCOS.

Characteristic	XIST		ETS2	
	R	*p* value	R	*p* value
BMI(Kg/m^2^)	0.186	0.5061	0.174	0.423
Age	− 0.013	0.62	0.014	0.71
LH (IU/L)	− 0.068	9.81	− 0.057	0.45
FSH (IU/L)	0.328	0.23	0.245	0.123
LH/FSH	0.1173	0.68	0.2224	0.723
AMH (ng/mL)	0.4056	0.19	0.568	0.236
T (nmol/L)	0.0564	0.0812	0.0469	0.0769
COC	− 0.253	0.2554	− 0.347	0.325
%MII	− 0.432	0.0446	− 0.456	0.0214
%GV	0.5113	0.0178	0.654	0.0389
%Degenerated.Oocyte	− 0.287	0.2063	0.954	0.478
%2PN	− 0.224	0.3273	− 0.221	0.659
%Blastocyst	0.0871	0.7073	− 0.123	0.84
%Cleavage	0.026	0.632	0.0491	0.758
Number of Useable.embryo	− 0.166	0.4579	0.347	0.589

*BMI* body mass index, *LH* luteinizing hormone, *FSH* follicle-stimulating hormone, *T* total testosterone, *AMH* anti-mullerian hormone, *COC* cumulus oocyte complex, *GV* germinal vesicle, *MII* Mature oocytes, 2PN fertilized oocytes with two primary pronucleus.

**Figure 8:**
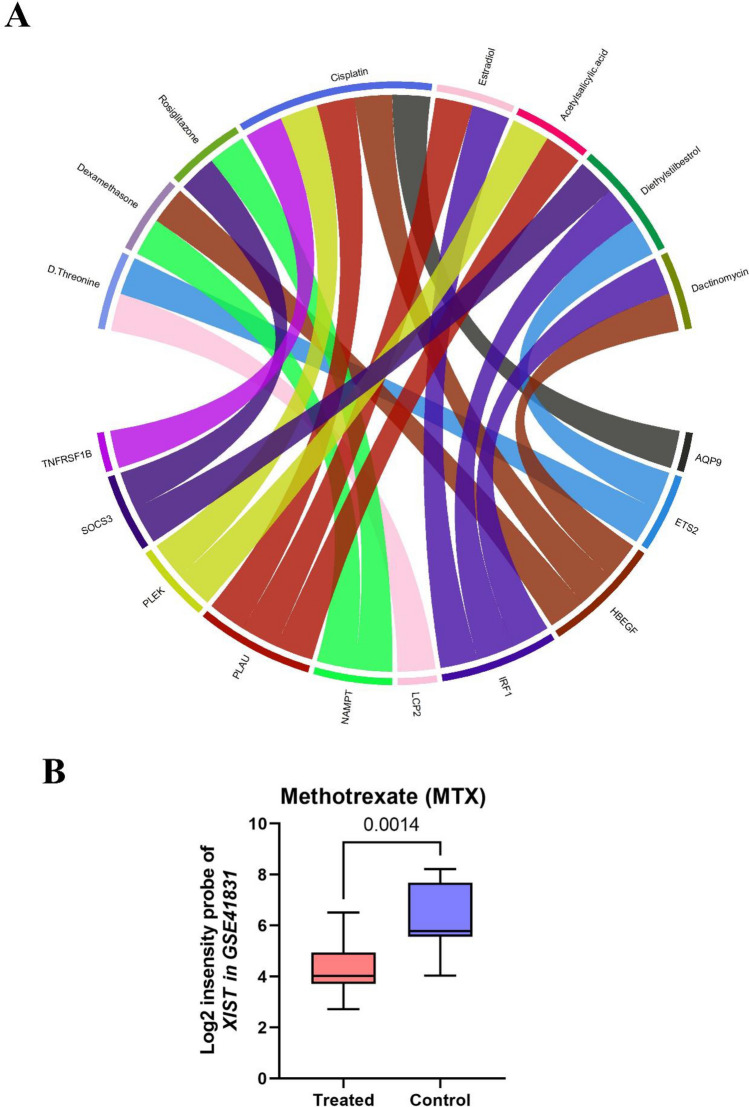
Identification of drugs that could target our candidate genes. (**A**) The Chord diagram represents flows or connections between several discovered drugs with candidate genes. (**B**) The expression level of *XIST* could be reduced by methotrexate significantly with FDR < 0.05. *XIST*, lncRNA X-inactive specific transcript.

The analysis of human drug discovery datasets revealed that certain drugs have the potential to simultaneously modulate the expression of multiple genes. For instance, D-threonine was found to target *ETS2* and *LCP2*, while dexamethasone exhibited the ability to target *NAMPT* and *HBEGF* genes. Additionally, our results suggested that rosiglitazone targets both *NAMPT* and *SOCS3*. Furthermore, the involvement of estradiol was observed in the regulation of *PLAU* and *IRF1* expression. Diethylstilbestrol was found to modulate the expression of *SOCS3*, *IRF1*, and *ETS2*. Lastly, acetylsalicylic acid exhibited an impact on *PLAU* and *PLEK* expression. (Fig. 8 A). Moreover, the analysis of GEO drug discovery showed that methotrexate could significantly decrease the expression of *XIST* in patients with juvenile idiopathic arthritis with an FDR < 0.05 (Fig. 8B). Hence, our analysis suggests that methotrexate and other identified drugs have the potential to serve as effective therapeutic agents for PCOS.

## Discussion

In the present study, we aimed to explore the underlying mechanism of *XIST* in PCOS, shedding light on new aspects of PCOS pathogenesis, and proposing novel medical compounds for its effective management.

LncRNA *XIST*, which is conserved among eutherians (human, mouse, rat, dog, cow, and elephant), is one of the most important lncRNAs that participates in the development of a wide range of diseases by acting as a competitive endogenous RNA (ceRNA)^16,17^. In the current study, the analysis of RNA-seq data showed a significant up-regulation of *XIST* in the GCs of individuals with PCOS. Furthermore, this over-expression of *XIST* in GCs of individuals with PCOS was confirmed by qRT–PCR. The results of public high-throughput analyses and qRT–PCR were quite different from those reported by Guo et al. who observed down-regulation of *XIST* in a human granulosa-like tumor cell line (KGN cells)^18^. Such a discrepancy could be attributed to the heterogeneous nature of PCOS. Moreover, it has been suggested that KGN cells might not be an ideal representation of PCOS, and to better mimic the PCOS cell model, it has been recommended to treat these cells with testosterone^19^.

Further validating the impact of *XIST*, we verified the up-regulation of 120 *XIST-*correlated genes in two separate cohort studies. Through enrichment analysis of these genes, we concluded that *XIST-*correlated genes were mainly enriched in various pathways. Notably, we focused on the genes enriched in inflammation-related pathways, including Inflammatory Response, IL-6/JAK/STAT3 and TNF-alpha signaling via NF-kB signaling pathways due to their fundamental roles in the pathogenesis of PCOS^15,20^.

To elucidate the potential regulatory mechanism of *XIST*, we identified miRNAs that exhibited a negative correlation with both *XIST* and *XIST-*correlated genes. Subsequently, a ceRNA network was constructed between *XIST* with 5 miRNAs including hsa-miR-1197, hsa-miR-193a-3p, hsa-miR-144-3p, hsa-miR-1271-5p and hsa-miR-146a-5p and 10 mRNAs including *AQP9, PLAU*, *ETS2, HBEGF, IRF1, LCP2, NAMPT, PLEK, SOCS3,* and *TNFRSF1B*. This is in agreement with previous findings, as it has been shown that up-regulation of *XIST* in various disease models, results in the sponging of four miRNAs, namely miR-27a-3p, miR-30c, miR-34a, and miR-146a, which in turn promote the inflammatory response^21–24^. Moreover, other studies have also reported the crucial roles of *XIST*- correlated genes in PCOS or PCOS-associated complications that are discussed below.

Aquaporin, AQP, is a water-specific membrane channel protein with 13 isoforms found in mammals (AQP0–AQP12)^25,26^. Three of these isoforms, AQP-7, AQP-8, and AQP-9 are expressed in GCs^27^. We demonstrated that *XIST* could influence the development of PCOS by stabilizing transcripts of *AQP9* through its function as hsa-miR-146a-5p, hsa-miR-1271-5p. It is of note that the expression of AQP-9 is known to be affected by fasting status as well as androgen levels. In the current study, the up-regulation of *AQP-9* in both GCs and blood of individuals with PCOS was observed across RNA-seq and microarray expression datasets. However, previous studies have reported no change^28^ or a decrease in *AQP9* expression in GCs of the PCOS group^29,30^. This discrepancy highlights the need for further studies to definitively confirm the extent of *AQP9* expression and its function in PCOS.

Plasminogen activator, urokinase (PLAU), is a member of the plasminogen system, which plays an essential role in ovulation and follicular angiogenesis^31,32^. Previous studies have reported higher levels of plasminogen activator inhibitor (PAI)-1 and lower plasmin levels in FF of PCOS women. However, to the best of our knowledge, there have been no published studies on *PLAU* mRNA levels in PCOS compared to non-PCOS women. In the present study, up-regulation of *PLAU* in both GCs and blood of PCOS samples was observed across 3 high-throughput datasets. This overexpression might be a compensatory response of GCs that promotes, through the *XIST*-hsa-miR-193a-3p- *PLAU* axis, to reduced plasminogen levels and the persistent increase in PAI-1 in FF of PCOS individuals^33–35^. Nevertheless, further experiments are needed to validate this hypothesis.

*ETS2,* as a member of the ETS family, is known to regulate genes involved in the cell cycle and apoptosis by functioning as a transcription factor. Consistent with the findings of this study, Jingyi Ren et al. recently showed that *ETS2* is up-regulated in PCOS individuals and PCOS mice models^36^. Furthermore, it’s worth noting that ETS2 has been reported to be overexpressed in breast carcinomas. The presence of *ETS2* binding sites on the *PLAU* promoter^37^suggestes that this transcription factor might be partially involved in the PCOS pathogenesis via *XIST*- hsa-miR-144-3p, hsa-miR-1271-5p and hsa-miR-146a-5p- *ETS2*- *PLAU* axis .

Heparin-binding EGF-like growth factor (HB-EGF) belongs to the epidermal growth factor family and is known to play essential roles in follicular development, ovulation, embryo implantation, and early development^38,39^. In support of our results, the up-regulation of *HB-EGF* in GCs and the elevated levels of HB-EGF in FF of PCOS individuals have been revealed previously. It appears that *HB-EGF* overexpression is achieved by sponging its regulatory miR, hsa-miR-1271-5p, by abnormally overexpressed lncRNA *XIST* in GCs. Consistent with our observations, Huang et al. reported that increased levels of HB-EGF in FF of PCOS individuals can enhance the apoptosis rate of GCs and lead to abnormal steroidogenesis through cAMP-PKA-JNK/ERK-Ca^2+^-FOXO1 pathway^40^.

Interferon regulatory factor 1 (*IRF1*) is the first member of the IRF family of transcription factors and is known for its role in activating the promoters of type I interferon genes^41^. It is proposed that *XIST*- miR-1197, hsa-miR-193a-3p- *IRF1* is another axis that is involved in PCOS pathogenesis. Interestingly, *IRF1* as the newly discovered PCOS candidate gene^42^; has also been implicated in the regulation of genes that are commonly associated with both type 2 diabetes (T2D) and PCOS such as *BIRC3* and *TNNL3*^43^*.*

Lymphocyte cytosolic protein 2 (LCP2) is a member of the SLP-76 family of adapters, which are critical intermediates in signal cascades downstream of various receptors^44^. Notably, *LCP2* is one of the major shared DEGs associated with both endometrial cancer and PCOS^45^. Furthermore, there is evidence suggesting a strong association between *LCP2* and ovarian cancer^46^. Our study revealed that overexpressed *XIST* interacts with both hsa-miR-144-3p, and hsa-miR-146a-5p as the competing endogenous RNA to increase *LCP2* expression in GCs of PCOS individuals.Nicotinamide phosphoribosyltransferase (*NAMPT*), also known as a visfatin, is a 52 kDa mammalian homodimeric adipokine^47^ that is expressed in several tissues, including GCs of the ovary^48–50^. *NAMPT* exhibits both intracellular enzymatic and extracellular cytokine-like activity, which facilitates its role in various processes such as energy metabolism, response to stress, inflammation, and insulin resistance^51^. In humans, plasma levels of *NAMPT* are increased during the progression of conditions such as obesity, T2D, and PCOS^52,53^. Considering the crucial role of *NAMPT* in modulating insulin sensitivity and its consequential impact on ovarian function, targeting and regulating the expression of this gene and its corresponding non-coding RNAs including *XIST*, hsa-miR-144-3p, and hsa-miR-1271-5p, offer a promising approach to alleviate the pathological symptoms associated with PCOS.

Pleckstrin, a 47-kDa protein resulting from the translation of the *PLEK* gene, was initially identified as a substrate of protein kinase C in platelets^54^. Subsequent investigations have proposed a variety of roles for this gene. Xie et al. suggested that *PLEK* may be involved in the development and maturation of human oocytes^55^. Additionally, Lu et al. reported a markedly elevated expression level of this gene in the adipose tissue of obese individuals in comparison to control subjects^56^. Considering the well-established association between obesity and PCOS, the potential effects of the *XIST*- hsa-miR-146a-5p- *PLEK* axis on PCOS characteristics merit further investigation.

The suppressor of cytokine signaling 3 (*SOCS3*) has been identified as a key negative regulator of the insulin signaling pathway. A growing body of evidence has highlighted the involvement of SOCS3 in the progression of insulin resistance^57^. In light of these findings, it is reasonable to hypothesize that elevated levels of *XIST* decoys hsa-miR-193a-3p to increase *SOCS3* expression in GCs. Overexpressed *SOCS3* contributes to the development of PCOS, partly by disrupting the insulin signaling pathway.

Numerous studies have reported elevated concentrations of inflammatory factors, such as tumor necrosis factor-a (TNF-a), and interleukin 6 (IL-6), in the serum and FF of infertile women with PCOS^58,59^. These findings reflect a low-grade inflammation that affects ovarian function, ovulation, fertilization, and implantation in this population. The actions of TNF are primarily mediated through two types of receptors: Type I (p55, p60, TNFAR, TNFR1) and Type II (p75, p80, TNFR1B or TNFR2) TNF receptors. In the present study, it was revealed that *TNFR1B* was significantly upregulated in GCs of PCOS individuals through the suppression of its regulatory miRNA, hsa-miR-193a-3p by *XIST*. In accordance with our results, studies have reported up-regulation of both TNF receptors in the adipose tissue of obese women. Furthermore, it has been proposed that polymorphism of the *TNFR2* gene (*TNFRSF1B*) may contribute to the pathogenesis of several metabolic disorders, including obesity and insulin resistance^60^. Interestingly, it has been reported that the methionine 196 arginine (676 T > G) variant in exon 6 of the *TNFRSF1B* is associated with PCOS and hyperandrogenism^61^.

We further assessed whether the expression of hub genes of our ceRNA regulatory network could be used as diagnostic markers in both GCs and blood samples from PCOS individuals. Our in silico finding revealed that *XIST* and all coding genes, except for *HBEGF9*, as well as 2 out of the 5 miRNAs, including hsa-miR-1197 and hsa-miR-193a-3p could potentially be used as diagnostic markers in GCs with a *p* vlaue < *0.05*. While 6 out of the 10 evaluated genes, namely *AQP9, ETS2, PLAU, PLEK, SOCS3,* and *TNFRSF1B* were identified as blood-based biomarkers.

In addition, a comprehensive review of our ceRNA network indicated that among the 10 evaluated mRNAs, only *ETS2* could be targeted by 3 candidate miRNAs, namely hsa-miR-146a-5p, hsa-miR-144-3p, and hsa-miR-1271-5p. Furthermore, enrichment analysis showed that this transcription factor could be enriched in the TNF-α signaling pathway^62^. Based on these findings, we hypothesized that *XIST*-hsa-miR-146a-5p, hsa-miR-144-3p, and hsa-miR-1271-5p-*ETS2* axis might play a pivotal role in the pathogenesis of PCOS. The observed increase in *ETS2* expression (Fig. 6M) provides further support for this hypothesis.

To further explore the relationship between *XIST* and *ETS2* and their role in the occurrence and progression of PCOS, we correlated the mRNA expression of these two genes with the clinical characteristics of PCOS individuals. We found that both *XIST* and *ETS2* were negatively correlated with the number of MII oocytes and positively correlated with the number of GV oocytes. These data suggested that *XIST* and its correlated gene *ETS2* might serve as potential therapeutic targets to effectively improve oocyte maturation processes in PCOS but how these effect are induced needs future exploration.

Finally, the identified diagnostic biomarkers were uploaded to online databases to explore potential drug molecules that could target these genes to treat PCOS and/or manage its symptoms. According to the GEO drug discovery, methotrexate was identified as a potential inhibitor targeting *XIST*. Methotrexate, formerly known as amethopterin, was initially developed for cancer treatment owing to its structural resemblance to folic acid and its ability to inhibit folate-dependent enzymes. It has also received FDA approval for the treatment of rheumatoid arthritis (RA). The therapeutic activity of methotrexate at the molecular level in RA involves the activation of multiple pathways that collectively contribute to the suppression of inflammation. One notable mechanism involves the inhibition of NF-κB activation by boosting adenosine release, promoting the activation of adenosine receptor A2A, and inhibiting the reduction of dihydrobiopterin to tetrahydrobiopterin^63^. Considering the well-established association between PCOS and chronic, low-grade inflammation, methotrexate emerges as a potential novel medication for managing PCOS. However, it is important to note that long-term low-dose methotrexate therapy may be associated with a wide range of side effects. To address this concern, simultaneous administration of methotrexate with folic acid is a viable approach and this possibility needs to be explored at least in animal models.

Among the other identified drug signatures, we focused on those that affected at least 2 out of 10 *XIST*-correlated genes, namely, cisplatin, dactinomycin, rosiglitazone, estradiol, diethylstilbestrol, threonine, dexamethasone, and acetylsalicylic acid. Among these drugs, cisplatin and dactinomycin were disregarded due to their severe side effects as anti-cancer drugs.

Interestingly in this study, rosiglitazone, a member of the thiazolidinedione drug family which is sometimes used off-label in PCOS management^64^, was identified as a candidate drug targeting both *NAMPT* and *SOCS3*.

Additionally, estradiol and diethylstilbestrol, a synthetic estrogen with higher bioactivity than estradiol, were also present among our candidate drugs. They were selected based on their ability to target *PLAU*, *IRF1, SOCS3*, and *ETS2*. This result aligns with the use of approved combined oral contraceptives, which often contain synthetic estrogens like ethinylestradiol, and are prescribed to alleviate PCOS-related hyperandrogenic skin symptoms such as acne and hirsutism^65,66^. In addition, Zhang et al. recently proposed diethylstilbestrol as a therapeutic option for T2D and PCOS based on bioinformatic analyses^43^.

Threonine, as an essential amino acid, plays a vital role in the TCA cycle. Interestingly, individuals with PCOS often exhibit disturbances of the TCA cycle. This is manifested by reduced citrate levels and elevated plasma levels of threonine, valine, phenylalanine, and tyrosine which in turn leads to a decrease in succinyl-CoA and fumarate^67^. The elevated levels of these amino acids could serve as a compensatory mechanism to provide cellular energy and counteract deficiencies in the TCA cycle activity. Consequently, threonine supplementation may be a viable consideration for the treatment of PCOS.

Dexamethasone is a glucocorticoid that was granted FDA approval for the treatment of various inflammatory conditions. The beneficial effects of dexamethasone have been demonstrated in clomiphene-resistant individuals with PCOS^68^. Furthermore, recent research has revealed that dexamethasone can partially ameliorate PCOS by reducing free testosterone and LH levels while enhancing follicular development. Dexamethasone has also been involved in clinical trials aimed at enhancing embryos and pregnancy rates in women with PCOS^69,70^. Although these studies report an association between dexamethasone and the improvement of PCOS symptoms, the underlying mechanism of dexamethasone is not yet clear. As part of the novel findings of this study, dexamethasone was introduced as a drug candidate that targets both *TNFRSF1B* and *NAMPT* for the management of PCOS.

Our results also suggested that acetylsalicylic acid, also known as aspirin, could have a positive impact on PCOS. Acetylsalicylic acid is classified among the nonsteroidal anti-inflammatory drugs. Given the anti-inflammatory effects of these agents, acetylsalicylic acid could participate in reducing PCOS-related inflammation pathways. In this regard, it has been recently reported that administration of low-dose aspirin combined with clomiphene citrate^71^, letrozole^72^, or tamoxifen^73^ is both safe and effective for PCOS, and improves pregnancy rate in this population.

This study has several limitations: (1) The study focused on expression levels of only *XIST* and *ETS2* in GCs among numerous coding and non-coding genes. (2) Due to the limited number of GCs obtained from control and PCOS individuals, a comparison of protein levels for the mentioned genes between these groups was not possible. (3) The proposed axis for the mechanism of *XIST* function in the pathogenesis of PCOS was solely based on bioinformatics analyses, and further wet lab experiments are required to confirm the accuracy of this data. (4) The study only suggested drug signatures for PCOS therapy based on a bioinformatic approach. However, further experimental verification and population-based studies are still needed to safely establish the clinical use of these agents.

### Conclusion

Due to the incomplete understanding of the pathogenesis of PCOS, there is currently no definitive cure for this condition. Therefore, it is crucial to shed light on new molecular aspects of PCOS pathogenesis to recommend specific medications. In this study, we demonstrated that aberrant expression of *XIST* contributes to the development of PCOS through the ceRNA network. Indeed, hub molecules of our ceRNA regulatory network were used to explore GC/ blood biomarkers and potential drug molecules targeting these genes to manage PCOS. Our results highlighted the use of methotrexate/folate and threonine as an important gene-targeting compounds for PCOS therapy. Overall, the findings of our study provide novel insights into the molecular etiology, diagnosis, and potential treatment strategies for PCOS. However, it is crucial to emphasize that further experimental validation is necessary to confirm these findings and translate them into clinical applications.

## Methods

### GEO data collection and processing

The RNA-seq count data of 6 granulosa cells (GCs) samples and miRNA-seq data of 10 GCs samples were obtained from the Gene Expression Omnibus (GEO) database (https://www.ncbi.nlm.nih.gov/geo/) through GSE138518 and GSE138572, respectively. Employing the “limma” and “edgeR” packages in the R software, genes and miRNAs with negligible or zero expression levels were excluded from the study. Subsequently, by implementing the TMM method, both data were normalized and converted into log2 format. The GSE34526 raw data files (.CEL format), run on an Affymetrix Human Genome U133 Plus 2.0 Array, were downloaded directly from GEO. The “limma” package in R and RMA method were executed for background correction, quantile normalization, and log2 conversion. This dataset comprises 7 PCOS and 3 control samples. To ensure data quality the “arrayQualityMetrics” package was employed for the quality assessment of each presentation. Samples that exhibited inadequate quality were subsequently excluded from the study. Furthermore, to corroborate the expression levels of the identified candidate genes in bloodsamples, the microarray dataset GSE54250 was downloaded from the GEO database. The GEOquery package was employed to obtain the normalized data, which was then used.

to assess the expression levels of our candidate genes in the blood sample. The detailed sample information of all studies is provided in Supplementary Table S1.

### Differential gene and miRNA expression analysis

Differential expression gene (DEG) analysis and differential analysis of miRNA expression (DemiRs) were conducted to identify and validate the differentially expressed genes and miRNAs between individuals with PCOS and the control group. Genes and miRNAs exhibiting *p value* < 0.05 and |logFC|≥ 1 and |logFC|< − 1 were considered candidate genes and miRNAs for downstream analysis and validation, respectively. Heatmaps and volcano plots of the DEG and DemiRs were generated using the “ggplot2” package within R software.

### Correlation test and enrichment analysis

To investigate genes associated with *XIST*, the Pearson correlation analysis was conducted between the expression levels of *XIST* and those of DEGs in 3 PCOS-GCs samples of GSE138518. A significance threshold of *p value* < 0.05 was applied. To visualize the *XIST*-associated genes, the Cytoscape 3.9.2 application was utilized. Furthermore, the Venn diagram tool was employed to validate the expression levels of genes between two cohorts (GSE138518 & GSE34526). Finally, to identify the *XIST*-related pathways, an enrichment analysis was carried out using the Enrichr tool and the Molecular Signatures Database (MSigDB) dataset. The significance level threshold was set at False Discovery Rate (FDR) < 0.05.

### Construction of the ceRNA network and

The miRcode database (http://www.mircode.org/) was used to predict *XIST*-targeted miRNAs. Interactions between candidate miRNAs and *XIST*-associated genes were established utilizing the TargetScan database. Genes exhibiting negative correlation patterns with miRNAs were included to construct the ceRNA network. To illustrate the interactions between *XIST* and *XIST-*correlated mRNAs, along with their corresponding miRNAs within the ceRNA network, the ggplot2 package in R software was used.

### Biomarker analysis in PCOS

In this study, Receiver Operating Characteristic (ROC) analyses were conducted using the pROC package to distinguish between two groups: PCOS and control individuals, based on gene expression levels. The diagnostic value of each gene was evaluated by calculating the area under the ROC curve (AUC). AUC values typically range from 0 to 1, with higher AUC values indicating better diagnostic performance.

### Drug discovery investigation

The identification and investigation of key genes play a pivotal role in drug discovery, as they provide valuable insights into potential drug targets and underlying mechanisms of action, ultimately leading to the development of more effective and targeted therapeutic interventions^74^. Discovering drugs that targeted our candidate genes was performed through several key steps. Initially, we utilized online drug discovery databases such as DGIdb (https://www.dgidb.org/), DrugBank (https://go.drugbank.com/), TTD (https://idrblab.net/ttd/), and GeneCards (https://www.genecards.org/). Each database was independently searched for the names of candidate genes and then the drug discovery approach was selected from the toolbar. This process yielded lists of drugs or compounds associated with each gene in the respective dataset. It's important to note that while these datasets are interconnected, certain information may not be uniformly present across all of them, and some drugs may be unique to a specific dataset. Notably, databases like DGIdb are connected to 18 other related databases. DGIdb in particular, retrieves target genes through 20 related databases^75^.

Subsequently, we focused our attention on drugs capable of targeting two or more candidate genes, thereby narrowing down our selection criteria.

Due to the absence of suggested drugs targeting lncRNA *XIST* in the mentioned databases, we used the GEO drug discovery database for transcriptomic drug screening using high-throughput methodologies. To identify a suitable study, we conducted keyword searches including the gene name, "*XIST*", "drug", and "treatment" on the GEO database (https://www.ncbi.nlm.nih.gov/geo/). Among the suggested studies, we selected the GSE41831 dataset. This dataset comprises 78 PBMC samples from healthy controls and juvenile idiopathic arthritis patients treated with methotrexate or methotrexate plus a TNF inhibitor. We downloaded the raw data from GSE41831 and conducted initial preprocessing steps. These steps involved background light removal, data normalization using the RMA method, and transformation of the data into a logarithmic scale. Finally, utilizing the normalized matrix, we assessed the effect of methotrexate on the expression levels of *XIST*. The outcomes were represented through a box plot. using a box plot.

### Meta-analysis validation: assessing the data

Raw RNA-Seq data from two independent high throughput cohorts, specifically GSE173160 with 6 PCOS and 6 control samples and GSE193123 with 3 PCOS and 3 control samples were acquired. Following normalization, the resulting normalized matrices from both cohorts were merged. To mitigate nonbiological technical biases such as batch effects, we employed the ComBat algorithm, as implemented in the sva package. For ensure comprehensive gene annotation, the GENCODE database (https://www.gencodegenes.org/) was employed for both lncRNA and mRNA. Subsequently, differential expression analysis was conducted, comparing samples from individuals with PCOS against control samples.

### Sample collection

In this study, 40 participants were recruited from individuals undergoing in vitro fertilization (IVF) or intracytoplasmic sperm injection (ICSI) procedures at the Pooyesh Fertility Center.The study received ethical approval from the Ethics Committee of the Royan Institute under the approval ID: IR.ACECR.ROYAN.REC.1400.103. Prior to the study, all participants signed informed consent forms. Diagnosis of PCOS women was carried out by licensed physicians according to the Rotterdam criteria, requiring the presence of two or more of the following criteria: polycystic ovary, oligomenorrhea/amenorrhea, and hyperandrogenism. According to the Rotterdam criteria, 20 women were diagnosed as having PCOS, with nearly 60% of them experiencing oligomenorrhea/amenorrhea. 20 control women exhibited no clinical or biochemical evidence of hyperandrogenism, lacked a history of endometriosis or other chronic diseases, and mainly applied for family balancing through Preimplantation Genetic Testing (PGT). Detailed clinical characteristics of all participants are shown in Table 3.

**Table 3:** Candidate drug and target genes based on the drug bank database (DrugBank, DGIdb, TTD, and GeneCards).

Drugs	Target genes	Drugs	Target genes
D-Threonine	LCP2, ETS2	Heparin	HBEGF
D-Tyrosine	LCP2	Cetuximab	HBEGF
D-Proline	LCP2	Panitumumab	HBEGF
Serine	LCP2	PM 102	HBEGF
Calcium	LCP2	KHK-286693	HBEGF
icotinamid	NAMPT	Copper	HBEGF
Nivolumb	NAMPT	Dextran	HBEGF
Niacin	NAMPT	Carboplatin	ETS2
Folic Acid	NAMPT	Gemcitabine	ETS2
Phosphoric Acid	NAMPT	D-Alanine	ETS2
Cholesterol	NAMPT, TNFRSF1B	Glycerin	AQP9
Dexamethasone	NAMPT, HBEGF	Urea	AQP9
Metformin	NAMPT	Fultamide	AQP9
Pioglitazone	NAMPT	Testosterone	AQP9
Rosiglitazone	NAMPT, SOCS3	Sorbitol	AQP9
Amiloride	PLAU	androstanolone	AQP9
Cisplatin	PLAU, PLEK, TNFRSF1B, HBEGF, AQP9	Teglarinad Chloride	NAMPT
Estradiol	PLAU, IRF1	Chs-828	NAMPT
Hydrocortisone	PLAU	Daporinad	NAMPT
Calcitriol	PLAU	Lithium	TNFRSF1B
Guanidine	PLAU	Stavudine	TNFRSF1B
Acetylsalicylic acid	PLAU, PLEK	Retinol	PLAU
Aprotinin	PLAU	Saruplase	PLAU
Azathioprine	PLAU	Razoxane	PLAU
Atorvastatin	PLEK	Alendronic Acid	PLAU
Framycetin	PLEK	Amediplase	PLAU
Indomethacin	PLEK	Camustine	PLAU
Inositol	PLEK	Hydrocortisone	PLAU
Cocaine	SOCS3	Filgrastim	PLAU
Ribavirin	SOCS3	Urokinase	PLAU
Nitric Oxide	SOCS3	Ionomycine	PLAU
Diethylstilbestrol	SOCS3, IRF1, ETS2	Verapamil	PLAU
Etanercept	TNFRSF1B	Upamostat	PLAU
Infliximab	TNFRSF1B	Dacarbazine	PLAU
Tasonermin	TNFRSF1B	Sirolimus	PLAU
Carbamazepine	TNFRSF1B	Bleomycin	PLAU
Didanosine	TNFRSF1B	Clodronic Acid	PLAU
Chloroquine	TNFRSF1B	Azacitidine	PLAU
Clozapine	TNFRSF1B	Iressa	PLAU
Curcumin	TNFRSF1B	Zoledronic Acid	PLAU
Chloramphenicol	IRF1	Papaverine	PLAU
Dactinomycin	IRF1, HBEGF	Geldanamycin	PLAU
Deferoxamine	IRF1	Celecoxib	PLAU

### Isolation of GCs

All eligible participants were treated with recombinant follicle stimulating hormone (FSH, Cinal F, Orchidpharmed, Iran) beginning on the second day of their last menstrual period and received gonadotropin-releasing hormone antagonist (Cetrotide, Merck, Germany) once the dominant follicle reached a size of 12–14 mm. When a minimum of three follicles with an average diameter of 16–18 mm were present, women were administered a dosage of Human Chorionic Gonadotropin (HCG) ranging from 5000 to 10,000 IU. 36 h following the administration of HCG, oocytes were collected under the guidance of vaginal ultrasound. Granulosa cells were extracted from follicular fluid (FF) following the previously described protocol^76^. Briefly, the FF along with the mural GCs were collected and transferred to a suitable falcon tube and centrifuged at 2000 rpm for 10 min. The clear yellow supernatant was discarded. The resultant pellet was suspended in Tyrode’s solution, and the suspension was transferred to the Sill-Select Gradient and centrifuged at 3000 rpm for 13 min. GCs were then isolated as a distinct white ring in the middle of the falcon tube and subjected to further centrifugation at 3000 rpm for 13 min. In the next step, red blood cell lysis buffer was added to the cell pellet and incubated for 5 min, serving to lyse and eliminate any remaining red blood cells from the gradient isolated cells. After centrifugation for 5 min at 1500 rpm, the solution was removed; and GCs were subjected to RNA extraction.

### Total RNA extraction, cDNA synthesis and RT-qPCR

Total RNA extraction from the samples was carried out using TRIzol (Yekta Tajhiz Azma, YT9064) following the manufacturer’s protocol. To ensure the removal of potential DNA contamination, the extracted RNA from each sample was treated with DNase( SinaClon, MO5401). Subsequently, cDNA synthesis was done by cDNA synthesis kit (Biotech Rabbit, BR0400403) in accordance with the manufacturer’s instructions. The *XIST* and *ETS2* specific primers were designed by Oligo 7 (USA) and purchased from TAG (Copenhagen A/S, Denmark). These sequences were as follows F:5′-CTTCCTCGTTGCTTACATCGG-3′and R:5′-ACTAGGGCATAATATCCCACT-3′, F: 5′-AGCGTCACCTACTGCTCTGTCA-3′, R: 5′-CGTTGCACATCCAGCAA-3′ for *XIST* and *ETS2*, respectively. To evaluate the expression levels, RT-qPCR was employed using SYBR Green PCR Master Mix (PCR biosystem, PB20.12-0.5), 10 pmol/µL of each primer, and 50 ng cDNA in a final volume of 20 µL for each reaction. *GAPDH* (F: *5*′-CCACTCCTCCACCTTTGACG-3 each primer, and 50 ng cDNA, R:* 5* each primer, and 50 ng cDNA - CCACCACCCTGTTGCTGTAG-3′) was used as an internal control. All measurements were carried out in triplicate, and data analysis was performed using the 2^−ΔCt^ method.

### Statistical analysis and use of softwares

The analysis and preprocessing of RNA-seq and microarray data were performed using the R programming language (v 4.2) with the latest package updates. All reported gene expression data consisted of three replications and is presented as mean ± standard error of the mean. Differences between individuals with PCOS and controls were assessed using an independent student's *t-*test, with statistical significance denoted by *p* < *0.05*. The Pearson correlation test was performed to investigate associations between variables. Data visualization and categorization were carried out using Cytoscape software (v 3.9) and GraphPad Prism (v 9).

### Ethics approval

The study received ethical approval from the Ethics Committee of the Royan Institute (IR.ACECR.ROYAN.REC.1400.103) and conducted in accordance with approved institutional guidelines.

### Supplementary Information

Supplementary Information 1.

Supplementary Information 2.

## Data Availability

The datasets generated during and/or analysed during the current study are available from the corresponding author on reasonable request.

## Abbreviations

*AQP9*: Aquaporin-9
AUC: Area under the ROC curve
ART: Assisted reproductive technologies
ceRNA: Competitive endogenous RNA
DEG: Differential expression gene
DEmiRs: Differential expression microRNAs
DGIdb: Drug Gene Interaction Database
ETS2: ETS proto-oncogene 2, transcription factor
FDR: False discovery rate
FF: Follicular fluid
FDA: Food and Drug Administration
GCs: Granulosa Cells
GEO: Gene expression omnibus
HCG: Human chorionic gonadotropin
*HBEGF*: Heparin binding EGF like growth factor
IVF: In vitro fertilization
ICSI: Intracytoplasmic sperm injection
*IRF1*: Interferon regulatory factor 1
LncRNA: Longe non-coding RNA
*LCP2*: Lymphocyte cytosolic protein 2
MRE: MicroRNA response element
MSigDB: Molecular signatures database
*XIST*: X-Inactive specific transcript
miRNA: MicroRNA
*NAMPT*: Nicotinamide phosphoribosyltransferase
PCOS: Polycystic ovary syndrome
PGT: Preimplantation genetic testing
*PLAU*: Plasminogen activator, urokinase
*PLEK*: Pleckstrin
PAI-1: Plasminogen activator inhibitor 1
ROC: Receiver operating characteristic
RA: Rheumatoid arthritis
*SOCS3*: Suppressor of cytokine signaling 3
SVA: Surrogate variable analysis
TTD: Therapeutic target database
*TNFRSF1B*: TNF receptor superfamily member 1B
T2D: Type 2 diabetes
