# Supplementary material for: Discovering therapeutic possibilities for polycystic ovary syndrome by targeting XIST and its associated ceRNA network through the analysis of transcriptome data
Source: Sci Rep. 2024 Mar 14;14:6180. doi: 10.1038/s41598-024-56524-1 (PMC10940664; doi:10.1038/s41598-024-56524-1)
Supplement: Supplementary file 2 — Supplementary Information 2. [file 41598_2024_56524_MOESM2_ESM.docx]

**Supplementary figure**

**Title:**  **Discovering therapeutic possibilities for polycystic ovary syndrome by targeting *XIST* and its associated ceRNA network through the analysis of transcriptome data**

Elahe Berenji1,2+ (M.Sc.), Ali Valipour Motlagh2+ (M.Sc.), Marziyeh Fathi1,2 (M.Sc.), Maryam Esmaeili2 (M.Sc.), Tayebeh Izadi2, Parsa Rezvanian2 (Ph.D.), Zahra Safaeinejad2† (Ph.D.), and Mohammad Hossein Nasr-Esfahani2† (Ph.D.)

1. ACECR Institute of higher Education (Isfahan branch), Isfahan, Iran

2. Department of Cellular Biotechnology, Cell Science Research Center, Royan Institute for Biotechnology, ACECR, Isfahan, Iran.

+ These authors contributed equally to this study.

†Co-corresponding Authors:

Zahra safaeinejad (safayinejad@yahoo.com)

Mohammad Hossein Nasr-Esfahani (mh.nasr-esfahani@royaninstitute.org)

Department of Cellular Biotechnology Cell Science Research Center, Royan Institute for Biotechnology, P.O. Box: 816513-1378, Isfahan, Iran.

Tel: +98 31 95015694, Fax: +98 31 95015687

**Figure S1: Differentially expressed genes (****DEGs) in granulosa cells (GCs) between PCOS and control individuals**
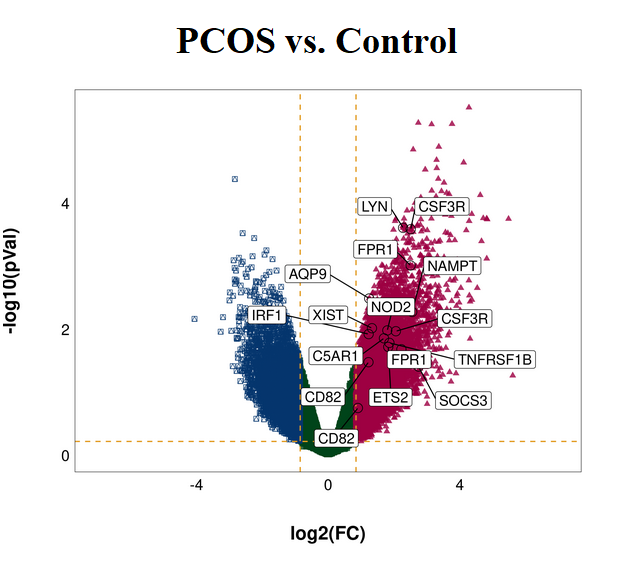


**Figure S2: The expression levels of 11 candidate genes in blood samples compared to control samples**


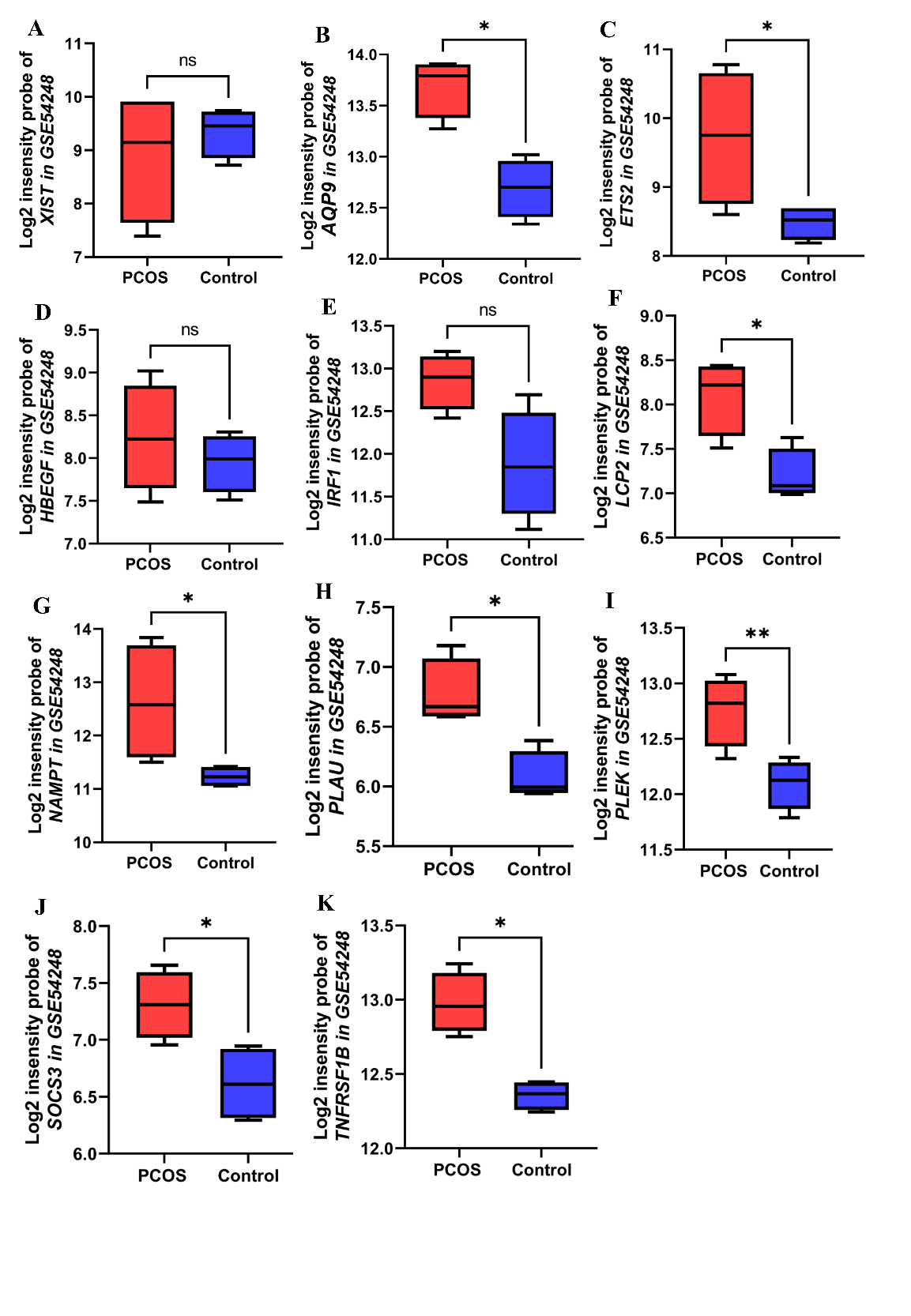


**Figure s3: Differentially expressed genes (DEGs) in granulosa cells (GCs) between PCOS and control individuals based on meta-analysis data**


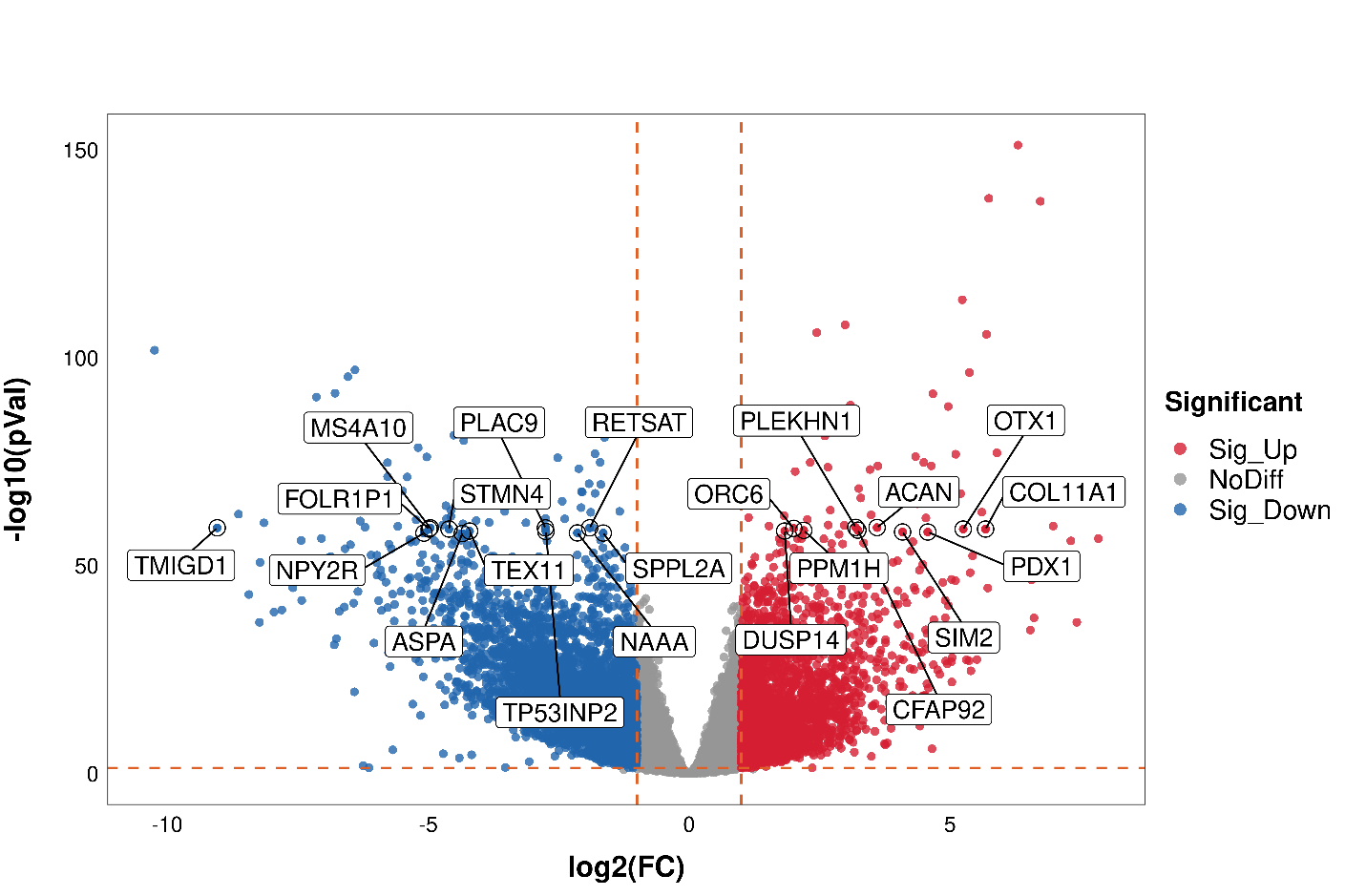


**Table S1: the study and sample information**

| **GSE study** | **Number of samples** | **Group** | **Data type** |
| --- | --- | --- | --- |
| GSE138518 | 6 | PCOS (GC) | RNA-seq |
| GSE138572 | 10 | PCOS (GC) | MicroRNA--seq |
| GSE34526 | 10 | PCOS (GC) | Microarray |
| GSE54248 | 8 | PCOS (Blood) | Microarray |
| GSE193123 | 6 | PCOS (GC) | RNA-seq |
| GSE173160 | 12 | PCOS (GC) | RNA-seq |

Polycystic ovary syndrome (PCOS)

Granulosa cells (GCs)

**Table S2: Enrichment analysis of top genes based on MSigDB dataset**

| **Term** | **Adjusted P-value** | **Combined Score** | **Genes** |
| --- | --- | --- | --- |
| Inflammatory Response | 2.18E-12 | 499.2716 | *LYN;CSF3R;CD82;AQP9;C5AR1;FPR1;NOD2;TNFRSF1B;LCK;IRF1;*  *NAMPT; NLRP3; FFAR2;LCP2;TLR2;HBEGF* |
| Allograft Rejection | 3.71E-09 | 283.8397 | *LYN;SPI1;STAT1;ITGB2;ITGAL;MMP9;FGR;PTPRC;LCK;NLRP3;*  *LCP2;LTB;TLR2* |
| Complement | 3.64E-07 | 180.7831 | *LYN;SERPINA1;ITGAM;SERPINB2;CR1;LCK;IRF1;PLEK;*  *CASP1;LCP2;PIK3CG* |
| IL-6/JAK/STAT3 Signaling | 4.18E-07 | 300.2016 | *SOCS3;CSF3R;STAT1;IRF1;LTB;TNFRSF1B;A2M;TLR2* |
| Coagulation | 1.18E-05 | 143.3111 | *THBD;SERPINA1;SERPINB2;PLAU;PLEK;PECAM1;A2M;MMP9* |
| TNF-alpha Signaling via NF-kB | 1.34E-05 | 105.3567 | *SOCS3;SERPINB2;PLAU;IRF1;NAMPT;PLEK;ETS2;TLR2;HBEGF* |
| Interferon Gamma Response | 1.34E-05 | 105.3567 | *SOCS3;STAT1;IRF1;NAMPT;FGL2;FPR1;CASP1;TXNIP;LCP2* |
| KRAS Signaling Up | 1.34E-05 | 105.3567 | *NIN;PLAU;ITGB2;PECAM1;CD37;LCP1;TNFRSF1B;MMP9;HBEGF* |
